# The Clinical Features and Progression of Late-Onset Versus Younger-Onset in an Adult Cohort of Huntington’s Disease Patients

**DOI:** 10.3233/JHD-200404

**Published:** 2020-10-08

**Authors:** Megha Anil, Sarah L. Mason, Roger A. Barker

**Affiliations:** Department of Clinical Neuroscience, John van Geest Centre for Brain Repair, University of Cambridge, Cambridge, UK

**Keywords:** Huntington’s disease, disease progression, age of onset, neurodegenerative disorders

## Abstract

**Background:**

Huntington’s disease (HD) is an autosomal dominant neurodegenerative disorder that typically manifests between the ages of 30 and 50 years. However, the disease can present at any age, and phenotypic differences between younger and later-onset patients have received limited attention.

**Objective:**

To compare clinical features of late- (>70 years of age) and younger-onset (<30 years of age) HD patients.

**Methods:**

Patients presenting to our regional NHS HD clinic with new-onset manifest HD diagnosed over the age of 70 years (LoHD) (*n* = 18) were compared with a younger cohort who developed disease under the age of 30 years (YoHD) (*n* = 12). Rate of progression over time on standard cognitive and motor measures was compared.

**Results:**

At first clinic presentation, both groups had the same total UHDRS scores. However, the LoHD group had higher chorea scores (F (1,28) = 6.52, *p* = 0.016), while the YoHD group had more dystonia (F (1,28) = 8.69, *p* = 0.006) and eye movement abnormalities (F (1,28) = 16.991, *p* < 0.001). The YoHD group also had a greater rate of motor progression, especially for bulbar measures (F (1, 28) = 6.96, *p* = 0.013) and bradykinesia (F (1, 28) = 7.99, *p* = 0.009). No differences were found in the rate of cognitive change (F (1,21) = 1.727, *p* = 0.203) nor functional capacity (F (1,28) = 1.388, *p* = 0.249) between the groups.

**Conclusion:**

Phenotypic differences between YoHD and LoHD patients were found in terms of initial presentation and rate of motor progression. This has implications for therapeutic trials involving HD patients of different ages, given their different clinical features and progression.

## Introduction

Huntington’s disease (HD) is an autosomal dominant neurodegenerative disorder that is characterised by a progressive decline in cognitive and motor function, as well as a range of psychiatric and other features. It is caused by a CAG expansion of the HTT gene at 4p16.3 [1], with clinical features appearing in all those with repeat lengths of 40 or greater, and to a lesser extent in those with repeats of 36– 39 where there is reduced penetrance.

The disease largely manifests in adults between the ages of 30 and 50 years, although it has been reported to present anywhere between 2 to 85 years of age [2]. Age at disease onset is closely correlated with the number of CAG repeats [3], but this only accounts for approximately 60% of the variance, with recent evidence pointing towards genes involved in DNA repair as also being important [4]. Increasingly, it is being recognised that there is a group of patients who present with clinical features at a much older age than is typical, the so-called late-onset Huntington’s disease (LoHD) patients. However, the definition for this group has been a matter of debate, with some using a cut off of 50 years, whilst others look at onset after the age of 60 years [5].

LoHD patients typically have a lower CAG repeat burden, with an average repeat length of 40.9 [5], but little else about the condition or its clinical features have been explored [6– 10]. Furthermore, while the juvenile variant of HD, in which features of the disease manifest before the age of 21, has been more intensely studied, with patients shown to have a much higher CAG repeat number (>55) and typically presenting with a more hypokinetic form of the disorder [2], little is known about patients with younger-onset adult disease (YoHD). We sought to compare YoHD and LoHD patients to investigate how the disease behaves at these extremes of age, which may inform about the disease pathogenesis more generally as well as having implications for therapeutic trials. We studied two cohorts of patients who attended our NHS HD clinic over the last 20 years, all of whom have been assessed by the same experienced neurologist (RAB). We selected patients for inclusion into the study who developed the disease over the age of 70 years (*n* = 18) or under the age of 30 years (a younger-onset cohort rather than JHD per se, due to the rarity of this latter condition) (*n* = 12).

## Materials and methods

**Fig.1:**
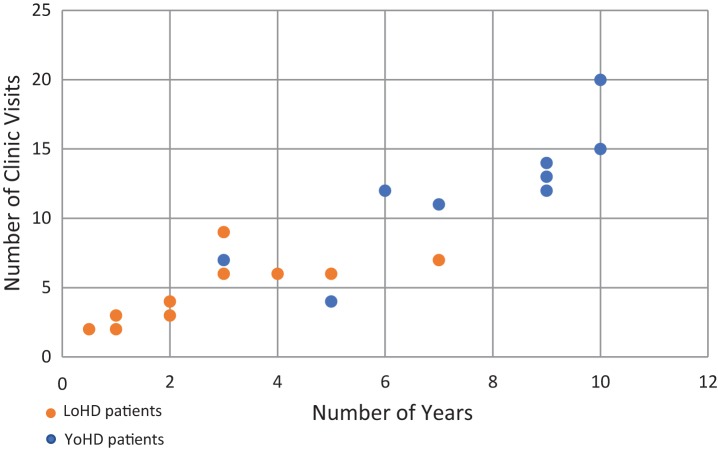
Scatter plot showing the number of visits a patient made to the HD clinic against the number of years over which those visits were made. Orange circles show LoHD patients, while blue circles show YoHD patients. The average number of visits made was 4.17 (*s* = 2.11) for LoHD patients and 9.75 (*s* = 5.40) for YoHD. These were made over an average period of 2.31 years (*s* = 1.71) for LoHD patients and 6 years (*s* = 3.37) for YoHD patients.

30 individuals were identified and included in our study from a retrospective dataset containing the results of the assessments collected as part of the standard care given to all patients attending the NHS HD clinic at the John Van Geest Centre for Brain Repair (VGB), Cambridge, UK. Cases were identified by the clinical team at the VGB because they had either been diagnosed with manifest HD after the age of 70 (LoHD) or before 30 years of age (YoHD). This age of onset was determined by the date at which clinical features were first noted either retrospectively by the individual or the family, if they had manifest disease at their first clinic visit, or prospectively by the clinician, if they were premanifest at their first clinic visit. All identifying features were removed from the data before being provided to the authors for blinded analysis, and as such the study was exempt from review by the local ethics board or NHS R & D department.

The LoHD group consisted of 18 individuals (12 female, 6 male), while there were 12 patients in the younger-onset group (6 female, 6 male). In the YoHD group, 2 patients had died at the time of data collection, whilst in the late-onset group, 1 patient had deceased and the living status of another patient was unknown. These patients were still included in the analysis.

Data collection for each patient visit started at the time of their first presentation at the clinic and continued until their latest clinic visit, which was determined by either their death, withdrawal from follow up, or end date for this study (July 2019).

Clinical data was then collected longitudinally from their first clinic appointment, for an average of 6 years (*s* = 3.37) for YoHD patients and 2.31 years for LoHD patients (Fig. 1).

The data collected included the total motor Unified Huntington’s Disease Rating Scale (UHDRS) score, which was then subdivided into specific motor elements by adding together different components of this scale (Table 1).

**Table 1:** Components of the UHDRS that were added together to form the different subgroups of motor features that were used in our analysis

Subgroup	Components of UHDRS used
(maximum score = 124)
Chorea	Chorea
*Dystonia*	Dystonia
*Luria*	Luria
*Bradykinesia*	Bradykinesia
Finger tapping (right and left)
Hand pronation (right and left)
*Bulbar Measures*	Speech
Tongue protrusion
*Eye Movements*	Ocular pursuit (horizontal and vertical)
Saccade initiation (horizontal and vertical)
Saccade velocity (horizontal and vertical)
*Gait*	Normal walk
Tandem walk
Pull test

As it was realised that most of the LoHD patients in this study would be retired, total functional capacity (TFC) scores were corrected by subtracting the employment component from all participants to give a maximum score of 10. Although the TFC score assesses the capacity to work rather than employment per se, it was felt that some jobs previously held by those in the LoHD group may now use different skill sets, such as electronic methods, that some patients may not be familiar with. In addition, ageing brings with it co-morbidities that could interfere with their capacity to work independent of any features of HD. Given all this, we felt that a more accurate way to compare the functional capacity of the two groups was by removing the employment element of the TFC entirely. In addition, we also used the UHDRS independence score in our analysis as a further measure of the functional impairment and level of HD-associated disability.

Cognitive function was determined using the Mini-Mental State Examination (MMSE), as this was the only cognitive test consistently collected across the groups, even though this test is known to lack sensitivity in HD due to its failure to assess executive function.

### Analysis

Differences in the total motor UHDRS score, the subgroup scores from the UHDRS, functional capacity, and the cognitive scores at initial presentation between the two groups were compared using a one-way analysis of variance (ANOVA). The rate of progression was calculated for these features and then a one-way ANOVA was also used to compare the rate of progression between the two groups.

**Fig.2:**
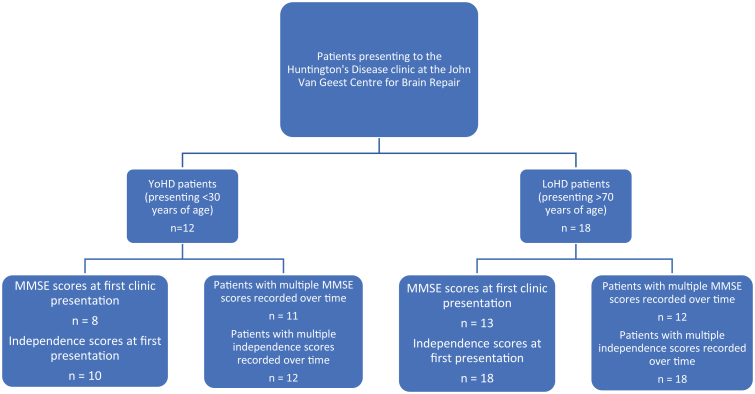
A flow chart showing the number of patients in the YoHD group and LoHD group for whom we had MMSE and independence scores at their first clinic presentation and the numbers where the rate of progression of MMSE and independence scores could be calculated. In the YoHD group, data for 8 patients was available for the analysis of MMSE scores at first clinic presentation whilst 11 patients were used in the analysis for the rate of progression of MMSE scores. Independence scores were available for 10 patients at presentation and 12 patients were used in the analysis for the rate of progression of independence scores. In the LoHD group, data for 13 patients was available for the analysis of MMSE scores at first clinic presentation, whilst 12 patients were used in the rate of progression of MMSE scores. Data from 18 patients were used in the analysis of independence scores in the LoHD group, both at first clinic presentation and over time. All other aspects of the motor UHDRS were recorded for all patients at all visits.

Five patients from the late-onset group and 4 patients from the younger-onset group had to be excluded from the cognitive analysis as no MMSE score was available for their first clinic visit. This left 13 patients in the LoHD group and 8 in the YoHD group to compare at their first clinic presentation. In addition to this, 2 patients from the younger-onset group had to be excluded from the independence score analysis as these values were not available for their first clinic visit. This left 10 patients in the YoHD group for this analysis (Fig. 2).

In contrast, 6 patients from the late-onset group and 1 patient from the younger-onset group had to be excluded from comparing the rate of cognitive progression, as they had only one MMSE assessment. Therefore, 12 patients in the LoHD and 11 patients in the YoHD group were used for this analysis (Fig. 2).

All analyses looking at the characteristics of disease onset were made using the assessments taken at first presentation for the YoHD group and those taken at time of diagnosis for the LoHD group.

All analyses were conducted using Microsoft Office Excel. p values less than 0.05 were considered statistically significant.

## Results

In the LoHD group, age at presentation ranged from 70 to 84 years, with a mean age of 77.3 years (*s* = 4.11). In the YoHD group, age at presentation ranged from 19 to 28 years, with a mean of 23.5 years (*s* = 2.43). There were statistically significant differences between the two groups in terms of CAG repeat length, with the average number of CAG repeats being 39.7 (*s* = 2.54) in the later-onset group and 59.4 (*s* = 5.48) in the younger-onset group (F (1, 25) = 167.534, *p* < 0.001).

### Motor features at disease onset

There was no significant difference between the total motor UHDRS score for both groups (F (1,28) = 3.68, *p* = 0.065). However, when we compared the scores of the individual components of the UHDRS between the two groups (Table 2) a statistically significant difference was found between age of onset and chorea (F (1,28) = 6.52, *p* = 0.016), dystonia (F (1,28) = 8.69, *p* = 0.006), and eye movement abnormalities (F (1,28) = 16.99, *p* < 0.001). The LoHD group had higher chorea scores, while dystonia and eye movement scores were higher in the YoHD group.

**Table 2:** The mean score of each subgroup of motor features at the initial presentation. With each subgroup, the mean score at initial presentation is shown, along with the 95% confidence intervals

Features	Younger-onset group			Late-onset group		
Mean score	95% Confidence intervals		Mean score	95% Confidence intervals	
Upper bound	Lower bound	Upper bound	Lower bound
UHDRS	33.25	42.39	24.11	23.67	30.01	17.33
Chorea	4.58	6.26	2.90	7.28	8.74	5.81
Dystonia	4.08	7.28	0.88	0.44	1.09	– 0.20
Luria	0.42	0.74	0.09	1	0.41	1.59
Bradykinesia	7.58	10.05	5.11	4.72	6.77	2.68
Bulbar measures	1.75	2.57	0.93	1.11	1.75	0.48
Eye movement	9.58	12.82	6.35	2.72	4.71	0.73
Gait	3.17	4.74	1.59	5.17	6.57	3.77

We found that there was no significant differences in the corrected functional capacity score at presentation (F (1,28) = 0.001, *p* = 0.975) between the two groups, nor in MMSE scores (F (1,19) = 0.23, *p* = 0.638). Independence scores also showed no significant difference at first presentation to the clinic (F (1,26) = 1.57, *p* = 0.221).

### Rate of disease progression

**Fig.3:**
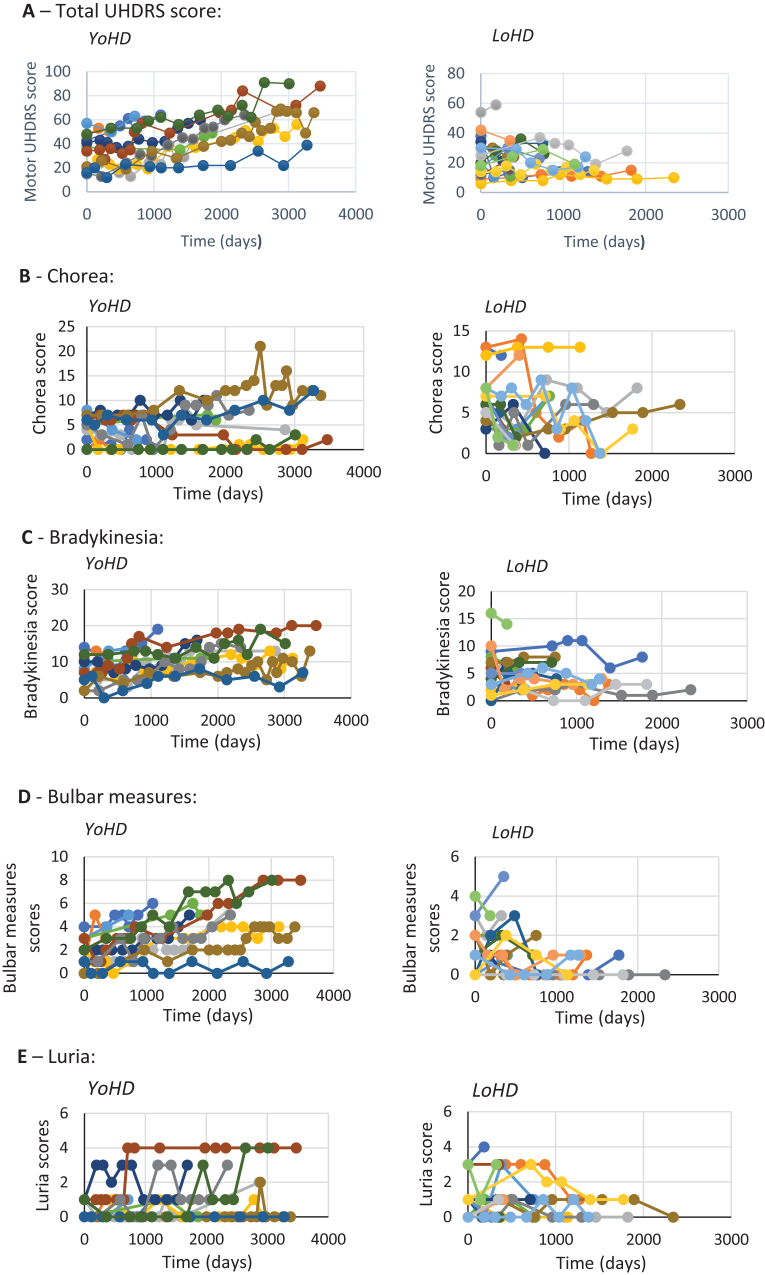
Graphs showing the progression of different features of HD over time. Each individual scatter line represents a single patient. A– E show the different features measured. Late-onset HD patients are shown on the right and younger-onset HD patients are shown on the left. A: rate of change of total motor UHDRS score (maximum possible UHDRS score = 124) B: rate of change of chorea (maximum possible chorea score = 28) C: rate of change of bradykinesia (maximum possible bradykinesia score = 20). D: rate of change of bulbar measures (maximum possible bulbar measures = 8) E: rate of change of Luria (maximum possible Luria score = 4).

Data from every clinic visit was plotted against time for each patient (Fig. 3) so that trends over time could be visualised between the age groups. In addition to this, an analysis of variance was undertaken to compare the rate of progression of disease features between the two groups (Table 3). This revealed there were significant differences for total UHDRS scores (F (1,28) = 7.14, *p* = 0.012), bulbar measures (F (1, 28) = 6.96, *p* = 0.013), and bradykinesia scores (F (1, 28) = 7.99, *p* = 0.009). These all progressed faster in the younger-onset group. No other statistically significant differences in motor functions were found between the groups.

**Table 3:** The mean rate of progression of each subgroup. With each subgroup, the mean rate of progression is shown, along with the 95% confidence intervals

Features	Younger-onset group			Late-onset group		
Mean score	95% Confidence intervals		Mean score	95% Confidence intervals	
Upper bound	Lower bound	Upper bound	Lower bound
UHDRS	1.27×10^–2^	1.57×10^–2^	9.76×10^–3^	– 4.58×10^–3^	6.35×10^–3^	– 1.55×10^–2^
Chorea	– 4.40×10^–4^	1.32×10^–3^	– 2.20×10^–3^	– 5.89×10^–3^	– 1.10×10^–3^	– 1.07×10^–2^
Dystonia	7.71×10^–4^	3.75×10^–3^	– 2.21×10^–3^	7.52×10^–4^	3.46×10^–3^	– 1.96×10^–3^
Luria	7.10×10^–4^	1.26×10^–3^	1.59×10^–4^	– 4.00×10^–4^	7.83×10^–4^	– 1.59×10^–3^
Bradykinesia	3.25×10^–3^	4.61×10^–3^	1.88×10^–4^	– 1.72×10^–3^	1.16×10^–3^	– 4.61×10^–3^
Bulbar measures	1.88×10^–3^	2.83×10^–3^	9.2×10^–4^	– 5.02×10^–6^	1.06×10^–3^	– 1.07×1
Eye movement	2.89×10^–3^	4.44×10^–3^	1.33×10^–3^	3.82×10^–3^	7.56×10^–3^	6.91×10^–5^
Gait	2.61×10^–3^	3.74×10^–3^	1.47×10^–4^	1.47×10^–4^	3.86×10^–3^	– 3.56×10^–3^

Cognitively, there was no significant difference in the rate of cognitive progression, as measured by MMSE (F (1,21) = 1.727, *p* = 0.203)— although the data on this was limited and this test is well known to be poor at predicting changes over time in HD.

The corrected functional capacity scores also showed no significant difference in their rate of progression over time (F (1,28) = 1.388, *p* = 0.249). Although this was corrected for presumed retirement in those >70 years old, TFC is a limited scale that lacks sensitivity due to having only 13 items. However, the independence score is a better reflection of progressive functional impairment, and so this was analysed and also showed no significant difference (F (1,28) = 0.353, *p* = 0.557).

## Discussion

In this study, we sought to compare the clinical presentation and then the motor and cognitive progression of a small cohort of patients diagnosed as having manifest HD after the age of 70 or before the age of 30 years at a single centre assessed by a single neurologist. Whilst we found no significant difference between UHDRS scores at first clinic presentation, the two groups had different predominant motor deficits at this time and progressed at different rates— the LoHD group had more chorea and a significantly slower rate of motor progression compared to the YoHD group who were more bradykinetic, with greater bulbar and eye movement abnormalities.

These results are consistent with an earlier study that found that those patients with a lower number of CAG repeats (which was what we found with our LoHD group, as one would expect) tend to have a more benign disease progression [11]. However, other studies have either found no difference in the rate of disease progression [12] or a greater rate of disease progression for those with LoHD [9]. This could relate to the fact that both of these studies compared LoHD with mid-age-onset HD, whereas, in our study, we looked at greater extremes of age, namely those with a younger age at presentation (<30 years old at presentation).

It is also interesting to note that differences in the rate of decline in motor scores were not uniform across the different components of the UHDRS, with bulbar measures and bradykinesia declining at a faster rate in the younger-onset group compared to the late-onset patients. In addition to this, there was a discrepancy between the components that differed between the two groups in terms of rate of progression versus those found at initial clinic presentation. Indeed, at initial clinic presentation, whilst no significant differences were found between the two groups for bulbar measures and bradykinesia, differences were found for chorea, dystonia, and eye movement; scores for dystonia and eye movement being higher in the younger-onset group, whilst chorea was higher in the late-onset group. The latter finding adds to the existing literature on the presentation of LoHD [5– 10, 13], although some these earlier studies [6, 11] were published when genetic testing for HD was not so precise and as such may not have been capturing the spectrum of disease accurately.

As a retrospective longitudinal study looking at two extremes of age at onset, there were aspects of it that we were unable to control. Polypharmacy was more common at first presentation to the clinic in the LoHD patient group, and this was partly a result of existing independent medical conditions. This could have confounded the results. In addition to this, in our study, clinical data was only available from their first presentation to our clinic, which is not the same as from diagnosis, given we had to use historical methods for ascertaining this in some patients, which is known to be somewhat unreliable. Linked to this is the possibility that the age at onset may have been recognised earlier in the YoHD patient group, as these patients may be more likely to have a positive family history, whilst the subtle initial manifestations of early HD may have been erroneously attributed to the effects of ageing in the LoHD population. However, a strength of this study is the consistency of assessment, as all of these were undertaken by the same experienced consultant neurologist at the clinic, which minimised inter-observer variance for this data.

Our study also showed no significant differences in cognitive features at onset and the rate at which they progressed. This is despite the well-known finding that there is an increased incidence of cognitive decline, and specifically, dementia with age in a non-HD population [14]. This perhaps reflects the milder clinical progression of LoHD compared with those that have a younger onset. This also raises the intriguing possibility that these LoHD patients have inherited or developed powerful compensatory processes for dealing with their proteinopathy. However, it should be stressed that the MMSE was the only cognitive test available to us in sufficient numbers to warrant analysis and it is known to lack sensitivity in HD, as it does not measure executive dysfunction and verbal fluency, two cognitive functions that are known to be majorly affected in HD [13]. Furthermore, several patients were excluded from the analysis due to missing data, leading to a very small sample size. All of this may account for the lack of significance. Further work is needed before any firm conclusions on cognitive change as a function of the age of disease onset can be made.

In conclusion, we have sought to better understand how HD presents and progresses as a function of age by using two extremes of disease onset. This would support the concept that earlier HD presents and progresses more rapidly around a hypokinetic disorder with marked bulbar and eye movement abnormalities. In contrast, LoHD is a more benign condition dominated by chorea and no major cognitive problems. As such, HD behaves differently depending on its age of onset and while we have looked at extremes of the age of disease onset, in reality, the condition will move from one phenotype to another as a function of any age of onset. A better understanding of this graded change in phenotypic expression and progression will be critical when we come to trial disease-modifying therapies for this disorder given their different dominant clinical features and the speed with which they progress with respect to these motor problems.

## Conflict of interest

The authors have no conflict of interest to report.
